# Advances in development and application of human organoids

**DOI:** 10.1007/s13205-021-02815-7

**Published:** 2021-05-08

**Authors:** Abhijith Shankaran, Keshava Prasad, Sima Chaudhari, Angela Brand, Kapaettu Satyamoorthy

**Affiliations:** 1 Department of Cell and Molecular Biology, Manipal School of Life Sciences, Manipal Academy of Higher Education, Planetarium Complex, Manipal, Karnataka 576104 India; 2 Department of Public Health Genomics, Manipal School of Life Sciences, Manipal Academy of Higher Education, Manipal, 576104 Karnataka India; 3 Department International Health, Faculty of Medicine, Health and Life Sciences, Maastricht University, Duboisdomein 30, 6229 GT Maastricht, The Netherlands; 4 United Nations University- Maastricht Economic and Social Research Institute On Innovation and Technology (UNU-MERIT), Boschstraat 24, 6211 AX Maastricht, The Netherlands

**Keywords:** Organoids, Stem cells, 3D culture, Growth factors, Disease modeling

## Abstract

Innumerable studies associated with cellular differentiation, tissue response and disease modeling have been conducted in two-dimensional (2D) culture systems or animal models. This has been invaluable in deciphering the normal and disease states in cell biology; the key shortcomings of it being suitability for translational or clinical correlations. The past decade has seen several major advances in organoid culture technologies and this has enhanced our understanding of mimicking organ reconstruction. The term organoid has generally been used to describe cellular aggregates derived from primary tissues or stem cells that can self-organize into organotypic structures. Organoids mimic the cellular microenvironment of tissues better than 2D cell culture systems and represent the tissue physiology. Human organoids of brain, thyroid, gastrointestinal, lung, cardiac, liver, pancreatic and kidney have been established from various diseases, healthy tissues and from pluripotent stem cells (PSCs). Advances in patient-derived organoid culture further provides a unique perspective from which treatment modalities can be personalized. In this review article, we have discussed the current strategies for establishing various types of organoids of ectodermal, endodermal and mesodermal origin. We have also discussed their applications in modeling human health and diseases (such as cancer, genetic, neurodegenerative and infectious diseases), applications in regenerative medicine and evolutionary studies.

## Introduction

Cell culture has been among the essential articulations used to study the mechanisms by which cells assemble into tissues and organs. Although two-dimensional (2D) cell culture is one of the major techniques through which researchers have elucidated a variety of aspects of cellular interactions, the uniform distribution of cells in the petri dish and the absence of nutrient gradients tend to diminish its physiological relevance (Bonnier et al. 2015). Thus, these conditions may not accurately recapitulate the in vivo architecture of cells and tissues (Bonnier et al. 2015). Increasing the dimensionality of the extracellular matrix (ECM) in a three-dimensional context will significantly impact cell survival, differentiation and proliferation.

Three-dimensional (3D) cell culture involves an artificially created growth environment where cells can interact in all three dimensions. 3D cell culture systems have special importance in fields such as drug discovery, as 2D culture models may not accurately reflect the response of a tissue to a particular compound (Joseph et al. 2019). Increasing evidence suggests that many promising drugs which had passed through the 2D monolayer screening process might have failed as the cellular microenvironment can alter response to the drugs (Hutchinson and Kirk 2011; Edmondson et al. 2014). Furthermore, tissues consist of multiple types of cells of diverse origin organized spatially and temporally along with the extracellular matrix (ECM); whereas many 2D culture systems generally involve multiple cell types with ECM often laid down by the cells itself or provided externally. As such, 3D cell culture models are more representative of in vivo tissue biology and may serve as a physiological model to study human diseases. One such 3D cell culture technique is organoid culture.

Organoids may be defined as cellular aggregates derived from primary tissues or stem cells that can self-organize into organotypic structures. Characteristically organoids comprise more than one cell type of the organ it represents; showcases physiological functions that are specific to that organ; and the cell organization similar to that of the organ itself (Stevens et al. 2009a). Frequently, organoids are grown in complex natural and artificial scaffolds, may show normal tissue architecture, and are highly heterogeneous. Therefore, each organoid generated is unique and exhibits random relative tissue positioning, possibly due to a lack of an embryonal axis for the cells to receive positional cues or signals (Lancaster et al. 2013). The heterogeneity in organoid cultures makes it difficult to obtain single-cell type cultures but has the potential to be an important method in drug development and diseases modeling at the organ level (Eiraku et al. 2011; Kondo and Inoue 2019).

**Fig. 1:**
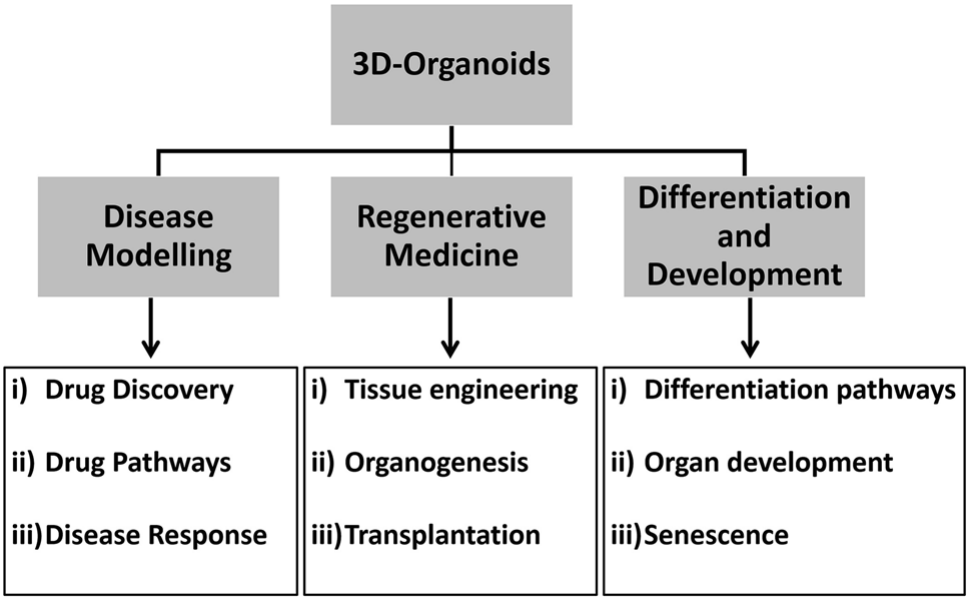
Various applications of 3D organoids in basic and translational research

Figure 1 outlines the applications of organoids in basic and translational research. For prospective drug development programs, cell-based assays are essential to assess the potential efficacy (Edmondson et al. 2014). 3D cell culture has been demonstrated to recapitulate in vivo tissue response more accurately than 2D culture. For example, tumoroids, which are 3D cancer models developed in vitro, demonstrate increased resistance to anticancer drugs (Loessner et al. 2010). This increased resistance of 3D models to treatment could be attributed to cell-ECM interaction, decreased drug penetration, and hypoxia, which are known to activate key genes promoting cell survival (Trédan et al. 2007). Further, organ-on-chip technology, which incorporates microfluidic systems into organoid culture, mimics vascularization and further recapitulating in vivo tissue architecture (Van Den Berg et al. 2019).

With advances in cellular reprogramming and personalized medicine strategies, one of the most exciting applications of organoids in disease modeling is biobanking. Organoid biobanks are collections of genetically and histologically characterized organoid models of disease states with matched controls collected from many individuals and this may contribute to advances in both basic and personalized medicine research. These biobanks have been especially valuable in modeling cancer (Schutgens and Clevers 2020). While this can be extended to a variety of disorders and conditions; however, till date, only one non-cancer organoid biobanks is reported which is the repository of intestinal organoid derived from cystic fibrosis subjects (Schutgens and Clevers 2020).

Thus, organoid cultures provide a robust platform through which numerous diseases can be studied; is a better physiological disease model in drug testing than 2D cell culture system and could also bridge the gap in drug response between cellular models and clinical trials (Kondo and Inoue 2019). Various advantages and disadvantages of 3D culture systems have been listed in Table 1.

**Table 1:** Advantages and disadvantages of organoid culture

Advantages
3D cell models are more physiologically relevant and predictive than 2D culture models The composition of the different cell types found in the organoid can be used to model the cellular interactions between different cell types within an organ By integrating microfluidics systems into the 3D culture, cellular response to “flow” (of blood or interstitial fluid) can be modeled Epithelial barrier tissues are known to separate the different compartments within an organ. There is a greatly enhanced representation of epithelium in organoids 3D cultures can be used to model inflammation better than 2D cultures Organoid culture can be initiated from ESCs, hiPSCs and ASCs
Disadvantages
Although physiologically organoids are close to in vivo organ systems, they lack vasculature and immune cells Most of the organoids are derived from iPSCs, the cells of PSCs are immature and match embryo/fetal gene expression profile Variability exists in many levels from genotypes of starting cells (iPSCs), to between batches, to within the batch between organoids, to within the organoid at different regions of organoid Creating a 3D scaffold that can accurately mimic cellular microenvironments is extremely intricate, especially the construction of the tissue-tissue interface Customizing the microenvironmental development factors that regulate the growth and differentiation of cells in vivo is challenging in in vitro 3D models Difficult to control the spatiotemporal distribution of nutrients and waste in these tissues

(References: Kim et al. 2020; Park et al. 2020; Pollen et al. 2019; Stevens et al. 2009a)

## Current organoid culture strategies

**Fig. 2:**
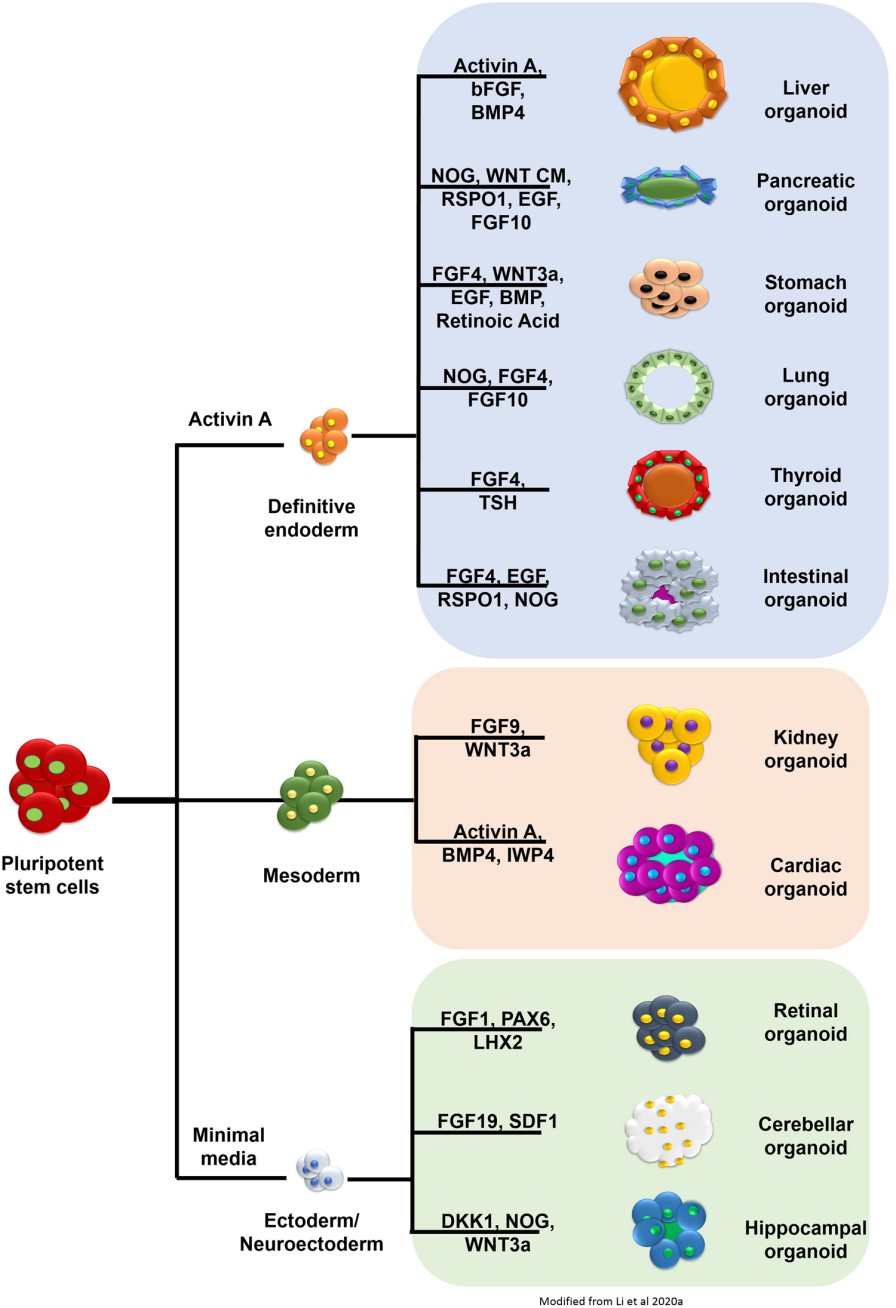
Brief overview of the various organoids generated along with the principal growth factors involved in the development of these organoids from pluripotent stem cells (Modified form Li et al. 2020a)

Organoids can be developed from pluripotent stem cells (PSCs), which can be embryonic (ESCs), induced (iPSCs), and adult stem cells (ASCs). Knowledge generated through the innumerable studies performed in the field of developmental biology have paved way for the establishment of protocols to culture various differentiated cell types from stem cell progenitors. Figure 2 indicates some of the key organoid models generated from PSCs and the principal growth factors involved in their generation.

### Ectoderm

#### Brain organoids

The central nervous system (CNS) of vertebrates is derived from the ectoderm after inductive signaling from the mesoderm. Su et al. (2006) pioneered the use of SFEBq (serum-free floating culture of embryoid bodies with quick reaggregation) in combination with bone morphogenic protein (BMP) and Wnt3a to induce embryonic stem cells (ESCs) to adopt a cerebellar fate. Eiraku and Sasai (2012) designed a SFEBq by placing the reaggregates in a minimal or serum-free media for a week, and then plating again in adhesion plates to promote self-aggregation of cells into spheroids. The ESCs then differentiate into a continuous neuroectoderm-like epithelium. This structure eventually forms stratified cortical tissues. These stratified tissues generally contain cortical progenitors, superficial-layered and deep-layered cortical neurons. When grown in media devoid of growth factors, the cells will spontaneously develop a hypothalamic fate. Other studies have shown that different regions of the brain can be generated by mimicking the endogenous growth factor patterning in vitro. Hedgehog signaling has been shown to drive ventralization of telencephalic progenitors/organoids developed from differentiation from ESCs that later form forebrain (Danjo et al. 2011). Lancaster et al. (2013) developed a method to generate “mini brains” or cerebral organoids containing representations of various regions of the brain. The method begins with embryoid bodies, and growth factors are not used to drive the differentiation of specific tissue lineages. The cells were embedded in Matrigel to allow for the growth of neuroepithelial buds, which in turn spontaneously give rise to various regions of the brain (Lancaster et al. 2013). Cakir et al. (2019) established a method to generate cortical organoids containing a vascular-like network by engineering hESCs to ectopically express ETS variant 2 (*ETV2*). These vascularized organoids acquired several blood–brain barrier characteristics and supported the formation of perfused blood vessels in vivo (Cakir et al. 2019). Pasca and co-workers generated 3D model in the absence of Matrigel which they called cortical spheroids. They developed brain organoids from embryoid bodies generated by PSCs maintained in non-adherent wells with high-dose ROCK inhibitor for 24 h. This was followed by neural induction using dorsomorphin and SB-431542 up to 5 days. Spheroids generated on the sixth day were then grown on neural media with FGF2 and EGF. These were differentiated into brain organoids comprising astrocytes and cortical neurons by the addition of brain-derived neurotropic factor (BDNF) and neurotropin 3 (NT3) (Pasca et al. 2015).

The human brain is sub-divided into three distinct regions: forebrain, midbrain and hindbrain. Forebrain, the largest of the three regions, comprises of epithalmus, hypothalamus, subthalamus, thalamus and cerebrum. Cerebral aqueduct, cerebral peduncle, tectum and tegmentum form the midbrain whereas the hindbrain comprises cerebellum, medulla oblongata and pons.

#### Forebrain organoids

Paşca and colleagues have also generated region-specific organoids that resembled the dorsal and ventral forebrain, called cortical and subpallial spheroids respectively, which showed a transition from fetal to postnatal phenotype at around the 280th day in vitro (Trevino et al. 2020). The method developed by Pasca and his team to generate forebrain organoids was built upon from the previously described protocols which involved the use of SMAD pathway inhibitors to induce neural differentiation in hPSCs followed by addition of ROCK inhibitors to aggregate the cells (Birey et al. 2017; Sloan et al. 2018). Briefly, hPSC colonies were lifted using enzyme dispase and cultured in non-adherent plates as suspension cultures. For the generation of dorsal forebrain spheroids, SMAD inhibitors, dorsomorphin (DM) and SB-4321542 (SB) were used for the initial 6 days; followed by addition of EGF and FGF2 growth factors up to 18 days. For maturation, BDNF and NT3 were added for the next 18 days. Ventral forebrain spheroids were generated in a similar fashion from hPSC colonies, altering the specific patterning cues toward ventral fate by adding WNT inhibitor (IWP-2), smoothened agonist (SAG), retinoic acid and allopregnanolone (Birey et al. 2017; Sloan et al. 2018).

Camp et al. (2015) discovered that cortical cells of cerebral organoids showcased gene expression profiles that very closely resembled to those of corresponding fetal tissue, indicating that organoids may recapitulate even gene expression patterns of their corresponding in vivo fetal tissue. Methods have also been developed to culture myelinated and unmyelinated neurons, astrocytes and oligodendrocytes of the cortex (Paşca et al. 2015; Marton et al. 2019).

##### Thalamic organoids

Thalamus helps in processing and relay of sensory information except for smell and mediating them to cortex. This organ is often associated with disorders and diseases related to movement, ataxia, visual loss, etc. Thalamic organoids were generated from the hESCs or iPSCs by differentiating to neuronal lineage using neural induction media containing SB431542 (Activin-like receptor kinase inhibitor), LDN193189 (BMP type I receptor inhibitor) and insulin for 8 days followed by thalamic patterning media containing BMP7 and PD325901 (MEK inhibitor) for another 8 days and subsequently grown in neural differentiation media (Xiang et al. 2020). In this method, thalamic organoids show representative markers such as TCF7L2 (Transcription Factor 7 Like 2), DBX1 (Developing Brain Homeobox Protein 1) and GBX2 (Gastrulation Brain Homeobox 2). Xiang et al. (2019) also showed that thalamus-like brain organoids in combination with cortical organoids can generate reciprocal projections between the two organoids.

##### Retinal organoids

The retina is a tissue that originates from the neuroectoderm and is the light receptive tissue of the eye. The optic vesicle originates from the diencephalon. The factors important for retinal differentiation include Paired Box 6 (PAX6), FGF1, FGF2, FGF9, PAX2 and Lim homeobox 2 (LHX2) (Heavner and Pevny 2012). Eiraku et al. (2011) grew murine ESCs in a serum-free medium to generate neuroectodermal cells. Matrigel was used to promote the generation of rigid neuroepithelial tissue. The neuroepithelial buds formed from this culture resembled primordial retinal tissue. These were then cut away and cultured in a medium that supports retinal development (Eiraku et al. 2011). The development of the organoid closely mimicked the processes that occur in the formation of the optic cup in vivo, showing proper apical-basal polarity and expressing various retinal and epithelial markers in a spatially correct manner. The organoids were large, and showed morphological features seen in human optic cups such as apical nuclear positioning and a higher proportion of rods and cones. Nakano et al. (2012) observed that the formation of photoreceptors in the organoids could be accelerated by Notch signaling. Völkner et al. (2016) then generated a protocol to generate 3D retinal organoids that do not require the evagination of optic vesicle-like structure, which until then had greatly limited the yield of optic organoids in culture. Chichagova et al. (2019) established a protocol for the development of retinal organoids from hPSCs. These organoids contained all the major retinal cell types and were responsive to light. The study involved the differentiation of hPSCs into retinal organoids via insulin-like growth factor-1 (IGF-1), retinoic acid and triiodothyronine signaling (Chichagova et al. 2019).

##### Hippocampus organoids

Located in the temporal lobe, the hippocampus has a major role in the learning and memory. Yu and colleagues developed a protocol for the generation of neuronal progenitor cells (NPCs) with hippocampal identity. Embryoid bodies were treated with antagonists of sonic hedgehog pathway and various factors that mimic the patterning of the forebrain, such as transforming growth factor- β (TGF-β) inhibitor SB431542, Dickkopf WNT signaling pathway inhibitor 1 (DKK-1) and Noggin (NOG). Hippocampal dentate gyrus-like granular neurons expressing PROX1 can be produced by treating the NPCs with high concentrations of WNT3a (Yu et al. 2014). Subsequently, hippocampal neurons were also generated by Sakaguchi et al. (2015). Using serum SFEBq as the starting material, they found that culturing cells with BMP4 and WNT agonist CHIR 99,021 induced formation of the choroid plexus. Changing the time window of exposure to these factors was found to induce self-organization of the tissue to form structures resembling the medial pallium. Formation of granule and pyramidal neurons was seen following long-term dissociation culture, both of which are found in the hippocampal region of the brain (Sakaguchi et al. 2015).

#### Midbrain organoids

Midbrain organoids are generated from embryoid bodies using hydrogels and orbital shakers embedding by sequential patterning or by simultaneous patterning method (Smits and Schwamborn 2020). In the sequential patterning, dual SMAD inhibitors (dorsomorphin, SB431542, LDN193189, A83, NOG) were used along with WNT modulation (CHIR99021) for the midbrain specification step, followed by midbrain floor plate induction using SHH modulation (smoothened agonist, SHH, purmorphamine) and FGF8, and finally, differentiation and maturation using neurotropic factors BDNF and brain-derived neurotropic factor (GDNF). In simultaneous patterning protocol, the midbrain specification and floor plate induction steps were combined together, followed by maturation step similar to sequential method but involving additional supplements (FGF20, TGFβ3, trichostatin A) (Smits and Schwamborn 2020; Galet et al. 2020).

#### Hindbrain organoids

Cerebellar development is controlled by the isthmic organizer, which is situated in the midbrain-hindbrain boundary. Muguruma et al. (2010) focused on the generation of purkinje cells, which are derived from the neuronal cells of the cerebellar ventricular zone. Using SFEBq, they combined insulin and fibroblast growth factor 2 (FGF2) to cause differentiation of the isthmic organizer, which secretes inductive factors leading to differentiation of cerebellar precursors (Muguruma et al. 2010). In another study by the same lab, it was reported that culturing human ESCs (hESCs) in the presence of FGF19 and stromal cell-derived factor-1 (SDF1) leads to the generation of structures that closely resemble the first trimester cerebellum (Muguruma et al. 2015).

#### Assembloids

Pasca (2019) described protocols for the generation of brain assembloids which involves mixing of various types of brain organoids or/and cluster of cells that later on synchronizes and organizes spatially (Pasca 2019; Vogt 2021). Three types of brain assembloids were a) multilineage assembloids b) multiregion assembloids c) polarized assembloids (Pasca 2019). Multilineage assembloids uses a combination of organoids of different cell lineages, such as a combination of neuronal cell lineage based spheroids with glial spheroids which may also have neuroimmune cells (microglia) and vasculature (pericytes and endothelial cells). Pasca and his team generated forebrain assembloids, (a multiregion assembloids) by growing together individual dorsal and ventral forebrain spheroids which eventually fuse leading to ventral forebrain spheroid cortical GABAergic neurons integrating with cortical glutamatergic neurons (Sloan et al. 2018; Pasca 2019). Recently, Cederquist et al. (2019) developed method to generate polarized assembloids that showed spatial topographic organization with latero-medial ganglionic eminences, hypothalamus, thalamus and dorsal forebrain regions. This was achieved by mixing together the forebrain organoids with Sonic Hedgehog (SHH) secreting cells. The Sonic Hedgehog gradient enables formation of positional axis in these polarized organoids (Cederquist et al. 2019; Miura and Pașca 2019).

### Endoderm

#### Stomach organoids

The stomach is a tissue that develops from the endoderm. Specifically, it develops from the posterior foregut of the gastrointestinal tract (GIT). McCracken et al. (2014) showed that temporal manipulation of FGF4, WNT3a, epidermal growth factor (EGF), BMP and retinoic acid pathways during 3D culture of hPSCs could be used to generate gastric organoids. They generated endodermal progenitor cells by treating hPSCs with activin. These cells were then differentiated into the foregut fate through addition of WNT3a, FGF4 activators and BMP inhibitors to the culture media. Addition of retinoic acid caused the organoids to move toward a posterior foregut identity. These tissues were converted into gastric organoids by the application of a high concentration of EGF and showcased molecular and morphogenetic development that mimicked the antrum of murine stomach. The organoids thus generated contained mucous cells, gastric glands and pit-like regions, Lgr5^+^ stem cells and many other endocrine cells of gastric origin. Stomach organoids can also be generated from multipotent adult stem cells (Watson et al. 2014). In the mouse stomach, Lgr5^+^ stem cells are located at the base of pyloric glands. Culture of these Lgr5^+^ stem cells generated long-term organoids that closely resembled the mature pyloric epithelium. Another marker used to identify stem cells in the stomach is Tumor necrosis factor receptor superfamily member 19 (TROY). *TROY*^+^ cells of human origin can be cultured in vitro to generate organoids resembling tissue from the corpus of the stomach (Stange et al. 2013). Broda et al. (2019) generated a protocol for generation of human antral and fundic gastric organoids from PSCs.

#### Small intestinal organoids

hPSCs can be differentiated into definitive endodermal cells upon treatment with activin A with 80% efficiency (D’Amour et al. 2005). FGF4 and WNT3a can then be used to promote differentiation of the endodermal cells toward mid/hindgut fates (Spence et al. 2011). The mid/hindgut spheroids, grown initially in a 2D culture system, were then cultured in Matrigel in the presence of EGF, R-spondin (RSPO1) and NOG. Over the next 1–3 months, the organoids expanded and grew to form polarized intestinal epithelium, containing structures that resembled villi and crypt-like regions of proliferation. The epithelia were surrounded by non-polarized caudal type homeobox 2 (CDX2)^+^ intestinal mesenchyme (Spence et al. 2011). Furthermore, transplantation of intestinal organoids generated from hESCs into mice resulted in the expansion and maturation of the epithelium and mesenchyme, which can be seen by the various differentiated intestinal lineages that were formed from the transplanted organoids (Barker et al. 2010). Recently, Kasendra et al. (2018) developed a method to generate primary human small intestine-on-a-chip from healthy regions of intestinal biopsies.

#### Lung organoids

The lung as an organ develops from the endodermal germ layer. Specifically, the lung, along with the thyroid, originate from the NKX2-1^+^ progenitor cells of the ventral foregut. NKX2-1 is a transcription factor that is vital for lung and thyroid development (Minoo et al. 1999). Longmire et al. (2012) established pure populations of Nkx2-1 progenitors that exhibit the differentiation capability of Nkx^+^ lung/thyroid tissue from mouse ESCs. Jain et al. (2015) established a protocol to culture cells of the distal airway from adult stem cells of *Pdgfra-H2B:GFP* mice. The alveolar organoids thus generated consisted of type I (gas exchanging) cells and type II (surfactant secreting) cells, but the culture conditions for these cells have not been completely defined, as co-culture with mouse lung fibroblasts is required for growth (Jain et al. 2015). Dye et al. (2015) devised a method to generate lung organoids from hPSCs. The hPSCs were directed into a definitive endodermal fate by Activin-A treatment. After differentiation into definitive endoderm, cells were incubated in foregut media with noggin, SB-431542 (inhibitor of the activin receptor-like kinase receptors), FGF4 and CHIR 99021 (WNT agonist). Spheroids were observed in the culture after 4 days and were transferred to Matrigel to facilitate 3D growth of cells. Simultaneous activation of hedgehog pathway specified the cells toward a lung fate. Mature lung organoids arose upon prolonged exposure to FGF10 in the Matrigel (Dye et al. 2015). Miller et al. (2019) developed a new protocol for the generation of lung organoids from hPSCs. The structures generated from their protocol resembled developing bronchi/bronchioles surrounded by lung mesenchyme, with cells that were expressing alveolar surface markers (Miller et al. 2019).

#### Thyroid organoids

The thyroid is an organ that develops from the same progenitor cells as the lungs during embryonic development (Minoo et al. 1999). Initial report suggested that transient overexpression of Pax8 and Nkx2-1 were enough to generate thyroid organoids from mouse ESCs (Antonica et al. 2012). The protocol established by Longmire et al. (2012) also generated thyroid organoids upon sequential exposure of progenitors to BMP/FGF4, followed by plating the cells in 3D architecture in Matrigel containing thyroid stimulating hormone (TSH) to induce development of thyroid organoids (Longmire et al. 2012). Building upon this protocol, Kurmann et al. (2015) established a protocol for generating thyroid organoids using mouse PSCs. The thyroid organoids thus produced and upon transplantation into hypothyroid mice, secreted thyroid hormones and rescued the mice with disease phenotype. Human thyroid organoids could be grown from iPSCs using the same protocol (Kurmann et al. 2015). Saito et al. (2018) reported a protocol for the long-term culture of thyroid organoids that was capable of iodide uptake and thyroglobulin synthesis. Transplantation of these organoids into mouse model of hypothyroidism resulted normal thyroid-like tissues (Saito et al. 2018).

#### Liver organoids

The liver is an organ whose progenitors arise from the foregut endoderm. It is perhaps among the most extensively researched tissues to create organoids due to several key applications (Prior et al. 2019). Human PSCs can be induced to form hepatic endodermal cells by treatment with activin-A, followed by bFGF/BMP4 treatment, in 2D culture. The hepatic cells thus derived from hPSCs were found along with endothelial cells and mesenchymal stem cells. When plated on Matrigel, spontaneous formation of 3D structures was observed. The liver organoids thus generated were vascularized and when transplanted into mice, the blood vessels in the organoids merged with the host vasculature within 48 h. Mice from lethal drug-induced liver toxicity were rescued by transplantation of liver buds generated in vitro (Takebe et al. 2013). Hepatic organoids could also be generated from Lgr5^+^ stem cells. The organoids could be dissociated and transferred to fresh matrix every 7–10 days for 6 months (Huch et al. 2015). Numerous tissue engineering methods have also been incorporated in the process of liver organoids production (Underhill and Khetani 2018). 3D-bioprinting strategies have been employed to generate liver organoids, with these showing higher liver function than monolayer counterparts (Ma et al. 2016; Norona et al. 2016). Pettinato et al. (2019) co-cultured human adipose microvascular endothelial cells with hiPSCs to produce liver organoids with greater differentiation yield and improved hepatic function across a wide range of parameters. Wang et al. (2019b) published a serum and feeder-free protocol for the establishment of hepatic organoids from ESCs. These cells did not produce teratomas when transplanted into fat pads of immune-deficient mice, enabled repopulation of the liver, and could be used to model alcoholic liver injury (Wang et al. 2019b).

#### Pancreatic organoids

As with the liver, pancreatic cells showcase an extremely low turnover rate and do not express Lgr5 under physiological conditions. However, pancreatic injury causes robust activation of the WNT signaling pathway and induces Lgr5 expression in the regenerating pancreatic ducts. Pancreatic progenitor organoids could be cultured from single pancreatic ductal cells, under modified mini-gut conditions. These organoids were able to expand fivefold weekly for over 40 weeks. These pancreatic progenitor cells were able to differentiate into ductal cells as well as endocrine cells upon transplantation (Huch et al. 2013). Ware et al. (2016) developed a protocol for the culture of pancreatic organoids using the hanging drop technique. Loomans et al. (2018) designed a protocol to expand adult pancreatic tissue into organoids containing pancreatic progenitor cells, with the potential to differentiate into endocrine tissue. The protocol for the generation of pancreatic organoids included pancreatic normal media that consisted of Noggin, WNT CM, FGF10, EGF, and R-spondin 3, prostaglandin E2 (PGE2) while the tumor media lacked EGF (Driehuis et al. 2020).

### Mesoderm

#### Cardiac organoids

Stevens et al. (2009a, b) developed a technique to generate cardiac patches of size 300–600 µm thick from cardiac cells obtained from differentiating hESCs. Briefly, human ESCs were differentiated into cardiomyocytes in Activin A and BMP-4 rich medium. These cardiomyocytes (CMs) were suspended on a rotary shaker. These patches comprised majorly cardiac cells (up to 80%) and primary epithelium. These aggregates of cells showed electromechanical coupling, but the patches did not survive post-transplantation (Stevens et al. 2009b). One of the reasons for short life of spheroids could be due to the fact that they did not match the heart tissue composition. Human heart contains about 70% of non-cardiomyocytes which are mainly cardiac endothelial cells for the supply oxygen as well as fatty acids to CM and cardiac fibroblasts for structure and maturation of CMs. To overcome the limitations, the cardiac patches comprising cardiomyocytes, endothelial cells and fibroblasts were generated. This second generation 3D patches showed greater stability, maturity and survival over two years after transplantation (Stevens et al. 2009a). Hudson et al. (2012) also generated primitive cardiac cells from hESCs, using a temporal differentiation protocol. Briefly, primitive streak was induced using BMP-4 and Activin-A, followed by inhibition of WNT signaling using small molecule inhibitor IWP-4 to induce cardiogenesis (Hudson et al. 2012). Ravenscroft et al. (2016) and Giacomelli et al. (2020) have found cardiac tri-cellular system better recapitulates the tissue physiology in vivo showing contractile maturity and enhanced inotropic response, with mature electrophysiology, mitochondrial respiration and sarcomere structures. In this protocol, hESC-derived CMs were used along with human primary cardiac microvascular endothelial cells (hCMECs) and human primary cardiac fibroblasts (hCFs) for the generation of cardiac tissue organoids and grown in non-adherent round bottom 96-well plates. Giacomelli et al. (2017) established protocol for cardiac organoids from pluripotent stem cells which followed simultaneous differentiation of CMs and endothelial cells after cardiac mesoderm induction. Pointon et al. (2017) used a high throughput imaging technique to study the effect of a large panel of inotropic compounds on these cardiac organoids with 80% sensitivity and 91% specificity.

Hinson et al. (2015) engineered cardiac tissues from dilated cardiomyopathy (DCM) patients to evaluate role of titin mutants in DCM that cause premature death by heart failure. Stable cardiac tissues were generated from hiPSCs of DCM patients and using RNAseq analyses. Hinson et al. (2015) identified pathogenicity of titin mutants. Richards et al. (2016) showed advanced strategies in developing biophysical microenvironment. In this protocol, investigators used silicon nanowires on hiPSC-derived cardiac spheroids and found increase in contractility, cell junction formation and reduction in spontaneous beat rate of the nanowired spheroids. This may be beneficial in reducing arrhythmic risk post-transplantation (Richards et al. 2016). Richards et al. (2017) developed a scaffold-free method to generate cardiac organoids using human iPSC-derived cardiomyocytes, human umbilical vein endothelial cells (HUVECs) and human cardiac ventricular fibroblasts. Cells were mixed relative to developing and adult heart. The developing heart ratio showed greater contraction amplitude and sarcomere formation (Richards et al. 2017). Hoang et al. (2018) generated cardiac organoids using 3D cardiac microchambers from 2D human iPSC colonies. In another study, Richards et al. (2020) developed a cardiac organ disease model for myocardial infarction (MI). In this model, cardiac microtissues fabricated in agarose hydrogel were placed in hypoxia chamber with 10% O_2_ and 1 µM noradrenaline up to 10 days to generate infarct organoids (Richards et al. 2020).

Tiburcy et al. (2017) developed an engineered human myocardium (EHM) for disease modeling such as heart failure and repair from ESCs and iPSCs under serum-free conditions. EHM was treated with norepinephrine hydrochloride and endothelin-1 along with ascorbic acid-2-phosphate sesquimagnesuim salt for 7 days to generate heart failure model (Tiburcy et al. 2017). Early maturation of iPSCs derived cardiac tissue can be conditioned to achieve adult human myocardium (Ronaldson-Bouchard et al. 2018, 2019a). In this protocol, the early stage hiPSCs cardiomyocytes (maturation at day 12 from spontaneous contractions) subjected to electromechanical conditioning and with increased induced contractions, resulting in the generation of cardiac tissue that closely recapitulated the adult human myocardium (Ronaldson-Bouchard et al. 2018, 2019a). In another protocol, the engineered cardiac tissue was subjected to increased electromechanical stimulation up to 21 days generating adult like phenotype (Ronaldson-Bouchard et al. 2019b).

#### Kidney organoids

The kidney originates from the embryonic intermediate mesoderm, which generates two progenitor cell populations, namely, the metanephric mesenchyme and the ureteric epithelium. These two cell populations form the nephrons and collecting ducts by interacting with one another. Initially, both these cell types could not be generated simultaneously, until Takasato and colleagues established a protocol to produce both the principle lineages of the kidney. Activin A and BMP4 were utilized to generate primitive streak identity in 2D culture human PSCs. FGF9 is then added to generate intermediate mesoderm identity. The cells then spontaneously developed into ureteric epithelium and metanephric mesenchyme and showed 3D structures when co-cultured with mouse kidney reaggregates or when cultured at extremely low densities in 2D. In a follow-up study, hPSCs were cultured in 2D with WNT signals for 4 days, after which they were treated with FGF9 for 3 days. These were cultured as organoids for up to 3 weeks. Brief exposure to WNT agonists greatly increased the number of nephrons seen in the organoid culture and results in the formation of kidney organoids with fully segmented nephrons, which were surrounded by renal endothelia and interstitium (Takasato et al. 2014, 2016). Morizane and Bonventre (2017) established a protocol for the generation of nephron progenitor cells with very high efficiency (80–90%) within 9 days of hPSC differentiation. More recently, Homan et al. (2019) developed protocols to culture kidney organoids in 3D-printed millifluidic chips. Their study showed that exposure of organoids to fluid shear stress (FSS) resulted in an enhanced peripheral expression of vascular markers melanoma cell adhesion molecule (MCAM) and platelet endothelial cell adhesion molecule (PECAM). They also showed a tenfold increase in the junctional density (number of branching points per unit area) and average vessel length (interjunctional distance) (Homan et al. 2019).

## Application of organoids in human health and disease

The rapid improvement in the methods for culture of patient-derived organoids has permitted disease modeling with greater precision suggesting their potential in translational medicine and personalized therapies. While methods for the development of organoids from PSCs had been established in the beginning of the twenty-first century, ASCs were thought to have limited replicative potential in vitro. One of the key advantages of using organoids to model diseases is that with the advent of iPSC-derived organoids, it is possible to personalize treatment based on the genome of individual patients. The generation of organoids from adult tissues require no genetic transduction, supporting the claim that organoids are a viable avenue for tissue transplantation (Laurent et al. 2011). The molecular identity and histology of organoids can be studied by a variety of techniques such as single-cell RNA sequencing and high-resolution microscopy techniques, such as electron microscopy and confocal microscopy (Stange et al. 2013; Freedman et al. 2015; Matano et al. 2015; Roerink et al. 2018). This allows for a much deeper understanding of stem cell biology, homeostasis in tissues and the pathophysiology of hereditary diseases such as cancer, cystic fibrosis, neurodegenerative and neuropsychiatric disorders and various bacterial and viral diseases (Bartfeld and Clevers 2015; Wells et al. 2016; Wang et al. 2020). Table 2 lists out the various diseases and conditions that the organoids have been used to model.

**Table 2:** Various organoid cultures, their source, and applications in disease modeling

Germ layer	Tissue	Source	Diseases modeled	References
Ectoderm	Brain	hiPSC, hESC, Tissue biopsies	Alzheimer’s disease, Autism spectrum disorders, Glioblastoma, Hypoxic brain injury, Lissencephaly, Microcephaly, Microlissencephaly, Miller-Dieker syndrome, Neuroblastoma, Parkinson’s disease, Sandhoff disease, Schizophrenia, Timothy syndrome, ZIKV-associated microcephaly	Allende et al. 2018; Amin and Paşca 2018; Bershteyn et al. 2017; Birey et al. 2017; Eiraku et al. 2008; Fusco et al. 2019; Gomes et al. 2020; Gonzalez et al. 2018; Iefremova et al. 2017; Jacob et al. 2020; Jin et al. 2017; Lancaster et al. 2013; Mariani et al. 2015; Pașca et al. 2019; Qian et al. 2017; Smits and Schwamborn 2020; Ye et al. 2017
	Retina	mESC, hESC, hiPSC	Enhanced S-Cone syndrome, Leber congenital amaurosis, Retinitis pigmentosa	Gao et al. 2020; Nakano et al. 2012; Perez-Lanzon et al. 2018; Völkner et al. 2016
	Inner ear	mESC, hESC, hiPSC	-	Jeong et al. 2018; Koehler et al. 2013
Mesoderm	Cardiac	hESC, hiPSC	Barth syndrome, Dilated cardiomyopathy	Hoang et al. 2018; Laflamme et al. 2007
	Kidney	hESC, hASC, hiPSC	Cancer, Polycystic kidney disease	Freedman et al. 2015; Guan et al. 2017; Takasato et al. 2014, 2016
Endoderm	Thyroid	mESC, hESC, hiPSC	Cancer	Antonica et al. 2012; Kurmann et al. 2015; Saito et al. 2018
	Lung	mESC, hESC, hiPSC	Bronchiolitis, Cystic Fibrosis, Hermansky–Pudlak syndrome, Idiopathic pulmonary fibrosis, Pulmonary tuberculosis	Chen et al. 2017; Desai et al. 2014; Li et al. 2020b; Longmire et al. 2012; Miller et al. 2019
	Liver	hESC, hiPSC, hASC	Alagille syndrome (ALGS), Cancer, Cystic fibrosis, Metabolic diseases	Guan et al. 2017; Huch et al. 2015; Takebe et al. 2013; Wu et al. 2019
	Stomach	hESC, hiPSC, hASC,	Cancer, Infectious diseases	Bartfeld and Clevers 2015; McCracken et al. 2014
	Pancreas	hESC, hiPSC, hASC	Cancer, Cystic fibrosis	Huang et al. 2015; Huch et al. 2013
	Intestine	hESC, hiPSC, hASC, Tissue biopsies	Cancer, Cystic fibrosis, Familial adenomatous polyposis (FAP), Hereditary multiple intestinal atresia, Inflammatory bowel disease	Angus et al. 2020; Dekkers et al. 2013; Sato et al. 2009

^*hASC* human adult stem cell, *hiPSC* human-induced pluripotent stem cells, *hESC* human embryonic stem cells, *mESC* mouse embryonic stem cells^

### Organoids as tools to model cancer

Cancer is a condition caused by mutation in cells that lead to their unregulated proliferation, invasion of surrounding and distant tissues through metastasis. The accumulation of mutations in cancer cells leads to considerable genetic heterogeneity within a tumor (Hanahan and Weinberg 2011). This tumor heterogeneity can also be a result of differences in epigenetic alterations, gene expression profiles, metabolic differences, metastatic potential and rates of proliferation of cells, and is considered the main reason for the failure of conventional cancer therapy (McGranahan and Swanton 2015; Fan et al. 2019). Of all the mutations in the cancer phenotype, the driver mutations cause cancer progression while, the drugs are often targets at passenger mutations that do not cause tumor progression (Stratton et al. 2009).

Models for studying cancer include cell lines, animal models and patient-derived tumor xenografts (PDTX), each of which has certain drawbacks that make it suboptimal for disease modeling (Dutta et al. 2017). Tumoroids are organoids that are derived from patient tumors and recapitulate the genotypic and phenotypic features of individual tumors, promoting personalization of therapy based on a patient’s genetic signatures (Sachs and Clevers 2014).

As proof of this concept, a tumoroid model was used to study pancreatic ductal adenocarcinoma (PDAC). The study used 5 PDAC tumoroid samples and treated them with an epigenetic drug, UNC1999, an inhibitor of histone methyltransferase, enhancer of zeste homolog 1 (EZH2) (H3 lysine 27 trimethylation writer). Out of the 5 tumoroids, the drug was effective against only 3 samples. Genetic analysis of the tumoroids revealed that only those 3 tumoroids retained the H3 lysine 27 trimethylation mark. This is indicative of tumoroids property of retaining the epigenetic signatures of the original tumor (Huang et al. 2015). Patient-derived organoids can also be used to model tumor microenvironments via co-culture with the cells that drive tumor microenvironment. Neal et al. (2018) established a protocol for an air–liquid interface culture of patient-derived organoids with immune and fibroblast components that can be grown from primary tumors. Furthermore, organoid technology, when combined with 4D-micropscopy, can be used to study cancer stem cell behavior in response to drug treatment to predict patient outcomes (Fatehullah et al. 2016). Tumoroids have also been shown to be good models to study hypoxia, oncogenesis, and metastasis (Saglam-Metiner et al. 2019). One of the major advantages of organoids in cancer research is it allows for precise individualized treatment strategies. The organoids from patient-derived healthy and tumor tissues can be comparatively evaluated for efficient drug screening. Certain cancers types such as gastric and gallbladder cancers are reported to have ascended from infectious agents. Co-culturing tumor organoid with infectious agent in vitro would help in understanding the mechanism of pathogenicity. Similarly, virus–cancer interactions; for instance, liver organoids co-cultured with hepatitis virus, or gastric organoids co-cultured with Epstein Barr virus can also be studied (Drost and Clevers 2018).

Jacob et al. (2020) established a glioblastoma organoid biobank from patient tissue and reported that the established organoids maintain parental tumor heterogeneity, mutation and gene expression. These organoids can be used to rapidly test patient-specific treatment strategies (Jacob et al. 2020). Recently, colonic organoids derived from patients with familial adenomatous polyposis (FAP) were used to test potential anticancer activity of XAV939, rapamycin and geneticin, and it was seen that geneticin specifically inhibited the growth of APC-mutant FAP-colonic organoids (Crespo et al. 2017). Numerous diseases, such as Huntington’s disease, Alagille Syndrome and several cancers have been modeled using organoids (Guan et al. 2017; Mehta et al. 2018; Tuveson and Clevers 2019).

Plasticity of cancer cells have not been entirely understood. A 4-Dimensional (4D) live cell imaging of organoids have been utilized to study the tissue dynamics and also lineage tracing. Intravital imaging involves stable fluorescent labeled organoid culture followed by its transplantation in vivo. This is followed by tracing and imaging the primary and metastasized cells of the labeled organoids in vivo up to one year (Rios and Clevers 2018).

### Modeling genetic disorders

The property of organoid culture to retain the genetic signatures of the original tissue makes it a reliable method to model genetic disorders. The current strategy for organoids for genetic disorders use either introducing mutations in the wild type organoids which uses CRISPR-Cas9 technology or establishing organoids from patient-derived samples or biopsies.

An example of a genetic disease that has been modeled using organoids is cystic fibrosis (CF). CF is an autosomal recessive condition that is caused by a mutation in the cystic fibrosis transmembrane conductance regulator (*CFTR*) gene (Riordan et al. 1989; Stutts et al. 1995). Over 2000 mutations have been identified within the *CFTR* gene, which results in a very wide distribution of severity of the disease phenotype. Dekkers et al. (2013) were the first to report the use of intestinal organoids to model CF. *CFTR* gene is activated by cyclic adenosine monophosphate (cAMP), the level of which is increased by forskolin, an activator of adenylyl cyclase. They developed a forskolin-induced swelling (FIS) assay that could be used to screen drugs against specific mutations of the *CFTR* gene to create personalized treatment modalities for CF patients. CF patient biobanks are now being developed with organoids generated from intestine, lung, kidney and pancreas (Dekkers et al. 2016; Hohwieler et al. 2017).

Multiple studies have been conducted in modeling and rescue of the retinal organoids in Retinitis pigmentosa (RP) caused due to either X-linked or autosomal mutations. Retinal organoids generated from patient-derived iPSCs have been used to study the tRNA nucleotidyl transferase, CCA-adding 1 (*TRNT1*) mutant in RP (Perez-Lanzon et al. 2018). In another study of X-linked RP mutation, the RP2 knockout and RP2 mutant patient-derived retinal organoids showed photoreceptor cell death on 150 days which was rescued using an adeno-associated virus mediated gene augmentation (Lane et al. 2020). Use of organoid technology in the repair and replacement of photoreceptors in RP has been detailed by Llonch et al. (2018).

Hereditary multiple intestinal atresia (HMIA) is an autosomal recessive genetic disease caused by mutation in *TTC7A* gene (tetratricopeptide repeat domain 7A). Intestinal organoids derived from two HMIA patients showed the pathogenicity of *TTC7A* mutations causing gain of function of differentiation and loss of function of epithelial proliferation (Bigorgne et al. 2014; Perez-Lanzon et al. 2018). Polycystic kidney disease (PKD) is a diverse genetic disease with two types of autosomal dominant and autosomal recessive with both causing formation of cysts in the kidney. Using CRISPR/Cas system, hESCs were gene edited to PKD mutant and kidney organoids were generated (Freedman et al. 2015; Perez-Lanzon et al. 2018). Timothy syndrome, the genetic disease, caused by mutations in calcium voltage-gated channel subunit alpha1 C (CACNA1C) gene, was studied in an assembloid model consisting of subpallium spheroids and cortical spheroids generated from patient-derived iPSCs. There was an observed reduction in saltation length of neurons that held back neuron migration. This was overcome by inhibiting the L-type calcium channel function (Birey et al. 2017).

Alagille syndrome (ALGS) is an autosomal genetic disease affecting multiorgan systems including liver, heart, skeleton and others. The liver organoid model generated from patients was used to study the pathogenicity of the mutant *JAG1* gene that encode Notch receptor ligand associated with ALGS. The organoid model study helped in the rescue of impaired ducts on reversal of *JAG1* mutation in vitro (Guan et al. 2017).

### Modeling infectious diseases

An organoid’s property of retaining the genetic signatures of the original tissue makes it an excellent model to study host-microbe interactions (Bartfeld 2016). Many of the pathogens that infect humans cannot be grown in 2D cultures. Due to advances in 3D cell culture and organoid techniques, microorganisms that were once “unculturable” are now amenable to culture. Examples of such infectious agents include norovirus and rotavirus. Both viruses are now cultured in either 3D or 3D-derived 2D culture systems. Hepatic organoids have been utilized by Baktash et al. (2018) to model Hepatitis C virus (HCV) entry into hepatocytes, demonstrating that HCV interacts with CD81, epidermal growth factor (EGF) receptor and scavenger receptor class B type 1 (SCARB1) and gathers in tight junctions, where it interacts with occludin and claudin-1 and enters the cell through endocytosis mediated by clathrin (Baktash et al. 2018).

A clinically relevant application of organoids in the study of host-microbe interactions is in the elucidation of diseases with high variation between individuals, due to various risk factors such as age, gender, and genetic risk factors. *Helicobacter pylori,* for example, infects about 50% of the human population, but only a small subset of individuals develops gastric cancer and gastric ulcers (Suerbaum and Michetti 2002). Organoid infected with *H. pylori* showcased increased proliferation due to oncogenic cagA and increased β-catenin signaling (Fatehullah et al. 2016). Establishing organoid biobanks of patients that developed gastric cancer due to *H. pylori* infection and studying the variations in gene expression in *H. pylori* molecular targets could provide a molecular basis for the differential response to *H. pylori*. Many other GIT infections have been modeled in organoids, including norovirus and rotavirus. Ettayebi et al. (2016) established that ex vivo enteroids support human norovirus infection. Human intestinal enteroids (HIEs) were grown as monolayers and infected with norovirus isolated from stool samples (Ettayebi et al. 2016). Rotavirus was successfully cultured by Saxena et al. (2016) by subjecting HIEs to lysates of MA104 cells infected with rotavirus. The HIE model maintained the virulence and supported replication of both norovirus and rotavirus. Yin et al. (2015) used HIEs to test the efficacy of an antiviral monoclonal antibody, which was found to significantly inhibit rotavirus infection.

3D organoids of the brain have also been used to study the pathogenesis of Zika virus (ZIKV) infection (Cugola et al. 2016; Dang et al. 2016; Qian et al. 2017). Neural organoid models were used to study the effect ZIKV infection had on neurogenesis and growth. Studies have shown that ZIKV infected neural stem cells (NSCs) show mitochondrial swelling, nuclear pyknosis, smooth membrane structures and viral envelops. These signatures were modeled in vitro*,* and infected organoids showed gene expression profiles that indicated that ZIKV prevented neurosphere formation during fetal development, leading to severe damage of tissues (Camp et al. 2015). Furthermore, ZIKV was found to induce the premature differentiation of infected NPCs and caused defects in the mitotic mechanisms in these cells and upregulate toll-like receptor 3 (TLR3), leading to defects in neurogenesis and eventually, cell death (Dang et al. 2016; Gabriel et al. 2017). Cerebral organoids can be models of neurodevelopmental disorders and have been used to show that previously approved drugs could inhibit zika virus replication and prevent vertical transmission of the virus (Zhou et al. 2017; Trujillo and Muotri 2018).

Kidney and liver organoids have even been used to model SARS-CoV-2 (Zhao et al. 2020; Allison 2020). The pathogenic mechanisms of many other microbes, such as *Toxoplasma gondii, Cryptosporidium* and *Salmonella*, have been modeled using organoids (Heo et al. 2018; Yin and Zhou 2018; Delgado Betancourt et al. 2019). Shigatoxigenic *Escherichia coli* is studied in PSC derived intestinal organoids. Similarly, pathogenicity and multidrug resistance *Mycobacterium tuberculosis* strain that causes pulmonary tuberculosis disease was evaluated in human lung alveolar organoids derived from adult stem cells. (Iakobachvili and Peters 2017; Li et al. 2020b). Based on the organoid study, cause of the gall bladder carcinoma was reported to be an infection with *Salmonella enterica* serovar Typhi. Study of other parasites such as *Nippostrongylus brasiliensis, Cyclospora* sp., *Cryptosporidium* sp. and *Giardia* sp. are also being evaluated (Iakobachvili and Peters 2017). *Listeria monocytogenes*, which causes food borne diarrhea in adults and meningitis in neonates is studied in intestinal organoids derived from fetal tissue. This bacterium rearranges the host cell actin to form a comet like tail formation. *Campylobacter jejuni* is studied in murine small intestinal organoids and *Clostridium difficile* is studied in induced human intestinal organoids (Hentschel et al. 2021). Pathophysiology of idiopathic pulmonary fibrosis that causes dyspnea due to fibrotic scarring in the lungs is modeled in lung organoids. Human lung organoids are also used to model respiratory viruses such as human parainfluenza virus 3 and respiratory syncytial virus (RSV) (Li et al. 2020b). Lung bud organoids are used in the modeling of Hermansky–Pudlak syndrome, bronchiolitis and pulmonary fibrosis (Chen et al. 2017).

### Modeling neurodegenerative and neuropsychiatric disorders

Organoids provide potentially viable alternative to understand the processes underlying the development and disorders of the human brain. The differentiation of neurons generated in vitro has been the main challenge in developing iPSC models for late-onset neurodegenerative diseases. Recent advances in culturing techniques have allowed for the growth of brain organoids from patients suffering from Alzheimer’s disease (Raja et al. 2016; Gonzalez et al. 2018). Gonzalez et al. (2018) reported the establishment of patient iPSC-derived cerebral organoids to study Alzheimer’s disease (AD). The study reported that the organoids, over time, spontaneously develop the biochemical and morphological traits associated with AD, including structures that very closely resemble amyloid plaques and neurofibrillary tangles (NFTs). These finding were unique to AD patient-derived organoids. Various controls, including iPSCs from healthy individuals, patients with Creutzfeldt-Jakob syndrome, mouse ESCs and mouse iPSCs did not share these cellular signatures (Gonzalez et al. 2018). Organoids have also been established from patients with Huntington’s disease. The phenotypes of neurons generated from patients were commensurate with the characteristics of neurons in vivo. iPSC-derived cortical neurons generated from patients with Huntington’s disease showed variation in gene expression, maturation and morphology, when compared to those derived from healthy individuals (Mehta et al. 2018).

Human organoids recapitulate the early developmental processes that occur in the brain of those affected by autism spectrum disorder (ASD) and could provide insight into its neurobiology (Mariani et al. 2015). Cerebral organoids from patients with ASD showed abnormal proliferation of neuronal progenitor cells and a higher number of GABAergic neurons. This finding supports the hypothesis that ASDs are caused by an imbalance in the excitatory/inhibitory neuronal networks in the brain (Rubenstein 2010). Trevino et al. (2020) recently used brain organoid models to understand the role of epigenetic modifications in the development of ASD. Their method showed that organoid models could be used to study mid-stage neurons as well as late stage (Trevino et al. 2020).

Mutations of the *DISC1* gene have been associated with various mood and neurodevelopmental disorders, such as depression, schizophrenia, bipolar disorder and ASD (Porteous et al. 2011; Prytkova and Brennand 2017). However, the precise mechanism by which *DISC1* mutations effect development of the brain has been unclear. Studies by Ye et al. (2017) indicated that DISC1 interacts with the protein NDE11 to regulate the attachment of NDE11 to the kinetochore. Mutations in the gene cause a disruption of DISC1/NDE11 complex formation, prolonging mitosis and thus affects cell cycle progression of glial cells (Ye et al. 2017). These findings were also mirrored in organoids established from patients with schizophrenia. Hippocampal dentate gyrus organoids that were established from these patients demonstrated reduced generation of granular neurons, decreased spontaneous neurotransmitter release and reduced neuronal activity, whereas those generated from bipolar patients showed hyperexcitability. The hyperexcitability of neurons generated from bipolar patients could be ameliorated by lithium administration only in those cells generated from patients who responded to lithium treatment (Mertens et al. 2015).

Parkinson’s disease (PD) is caused due to accumulation of Lewy bodies that lead to selective degeneration of dopaminergic neurons. PD can be studied by modeling of midbrain organoids (Smits and Schwamborn 2020; Galet et al. 2020). Animal models used to study the brain development and function in neuropsychiatric diseases fail to match the human brain system. Human brain organoids serve as a better model in such cases. For example, the effect of prenatal drug exposure in neocortical organoids such as cocaine showed premature differentiation and neurodevelopmental defects due CYP3A5-induced reactive oxygen species (ROS) production. This study can be extrapolated to evaluate the targets and effects of drug abuse on brain (Lee et al. 2017; Wang 2018).

### Applications in regenerative medicine

One of the major therapeutic applications of organoids is in the field of regenerative medicine. Autologous organoid transplantation is an exciting application for organoids. Organ transplantation therapies carry risks of rejection and shortage of suitable healthy organs can adversely affect patient treatment (Prior et al. 2019). Organoid has been identified as promising alternative to small intestine, liver, kidney, or other tissue’s transplantation (Sato et al. 2009; Huch et al. 2015; Kim et al. 2018; Mansour et al. 2018). ASCs from the healthy region of the affected organ have the potential to differentiate into an organ tissue. This characteristic of ASCs can be used in the repair and transplantation of grafts (Drost and Clevers 2017). Organoids can serve as an alternative for organ transplants in two ways, a) as a replacement in diseases and injuries such as retinal degeneration or intestinal damage and b) as replacement for gene defective organs through gene-corrected organoids (Lancaster and Knoblich 2014).

Kidney organoids developed from patient-derived PSCs have been studied as a potential alternative in kidney transplantation (Grassi et al. 2019). Transplantation of intestinal organoids generated from intestinal stem cells through endoscopy could help patients recover from intestinal bowel disease (Okamoto et al. 2020). Human islet-like organoids generated by Yoshihara and team showed recovery of glucose homeostasis after transplantation in diabetic NOD/SCID mice (Clarke et al. 2018). Engraftment of cerebral organoids into mouse models showed enhanced survival and vascularization compared to transplants of neural progenitor cells. The transplanted organoids contained a large NSC pool and showcased multilineage neurodifferentiation at 2 and 4 weeks post-grafting (Daviaud et al. 2018). Lenti et al. (2019) recently generated lympho-organoids that contained T and B cells which support antigen-specific immunity upon transplantation into mice.

Furthermore, organoid models may play a major role in precision medicine, with diseases affected various organs could be modeled with the patients’ genetic signatures (Lenti et al. 2019). Once the organoids are generated with patients’ genetic signatures, such organoids can be tested for effective drugs for corresponding disease or disorders. This make the treatment module precise and personalized. Ivacaftor drug tested in organoids generated from CF patients, showed promising results and later translating into those patients helped recuperate (Takahashi 2019). Similarly in pancreatic duct cancer, drug screening using organoids generated from patients has been found to be promising in discovering effective drug (Takahashi 2019). Li et al. (2020a, b) has listed a number of cancers where in patient-derived organoids were used in the screening of anticancer drug that are effective and less toxic. In recent years, advances in the translational research has made it possible to generate and cryopreserve organoids from patient-derived tumor and normal cells in organoid banks (Li et al. 2020a).

## Lab-on-chip platforms

As organoids begin to grow, the core of each organoids becomes more distant from the nutrient media, causing lack of nutrition. Furthermore, the buildup of waste that is not removed also contributes to death of cells in the core of the organoids (Sailon et al. 2009). These limitations can be addressed using microfluidic platforms, called organs-on-chip or lab-on-chip, to deliver nutrition to the center of each organoid and remove the waste generated at the core. In a study by Karzbrun et al. (2018), the authors used the lab-on-chip brain organoid model to study the wrinkling and folding mechanism during brain development. Brain organoid-on-chip models have also been used to study of effect of prenatal nicotine and cannabis exposure on brain development (Wang et al. 2018; Ao et al. 2020).

Another field in which organ-on-chip models have shown significant potential is toxicology where it has been suggested that these models may help to reduce the number of animals used and to potentially replace animal models (Marx et al. 2016). Existing preclinical models of toxicology are ineffective, with only 12% of candidate drugs from clinical trials reaching the market. Animal models vary in blood flow, transporter expression and plasma protein binding, thus, tubular secretion data cannot be extrapolated to humans. Ex vivo tissues have limited viability with 2D monocultures are viable only for 2 weeks and interindividual variability leads to errors in generalization (Yeung and Himmelfarb 2019). Thus, robust and predictable preclinical models of the renal tubules are required to study secretion, absorption and infection. Sakolish et al. (2018) used a kidney-on-chip models in combination with an in silico model that extrapolates results to in vivo clearance and predicts renal clearance using creatinine (negative control), perfluorooctanoic acid (positive control), gentamycin and cisplatin. Although the model does have drawbacks that need to be addressed, it provides fairly accurate predictions for tubular reabsorption (Sakolish et al. 2018). Wang et al. (2019a) utilized kidney-on-chip to study virus-related kidney dysfunction, using a pseudorabies virus induced kidney disease model. The distal tubular model showed dysregulated Na^+^ reabsorption, broken reabsorption barriers and transformed microvilli, demonstrating that lab-on-chip platforms can be used to understand the pathophysiology of diseases (Wang et al. 2019a).

Thus, organoid-on-chip models may provide a way through which some of the drawbacks of traditional organoid culture (TOC) can be addressed. TOCs fail to recapitulate the precise microenvironment and its cues for organ development in vivo. One such area where organ-on-chip can help improvise the organoids is in the management of biochemical microenvironment. In TOCs controlling the morphogen gradients at different time points becomes a challenging task. The organ-on-chip model uses a pair of microchannels that can act as a source and sink to precisely control the biochemical environment (Park et al. 2019). This model was use in the development of neural tube patterning where SHH signaling and BMP gradients are formed along the dorsoventral axis (Park et al. 2019). Similarly, this model was used in generating intestine-on-chip by creating a WNT and BMP signaling molecule gradients in the intestinal organoids (Park et al. 2019). Lack of vasculature in the traditional organoid culture cause two problems: a) oxygen supply and b) nutrient supply and waste removal from the inner core of the organoids. Organ-on-chip model with the microfluidic device that has dual microchannels that can serve as an ideal alternative to vasculature found in organs in vivo. Biophysical environment, another challenge in the traditional organoid culture, is overcome using organ-on-chip model. For instance, the peristalsis or the movement of gut can be mimicked using a peristaltic pump attached in a stomach-on-chip model. Yet another use of organ-on-chip platforms is in reducing variability. The automated system help reduce batch to batch variability in size, structure and gene expression patterns (Park et al. 2019).

**Fig. 3:**
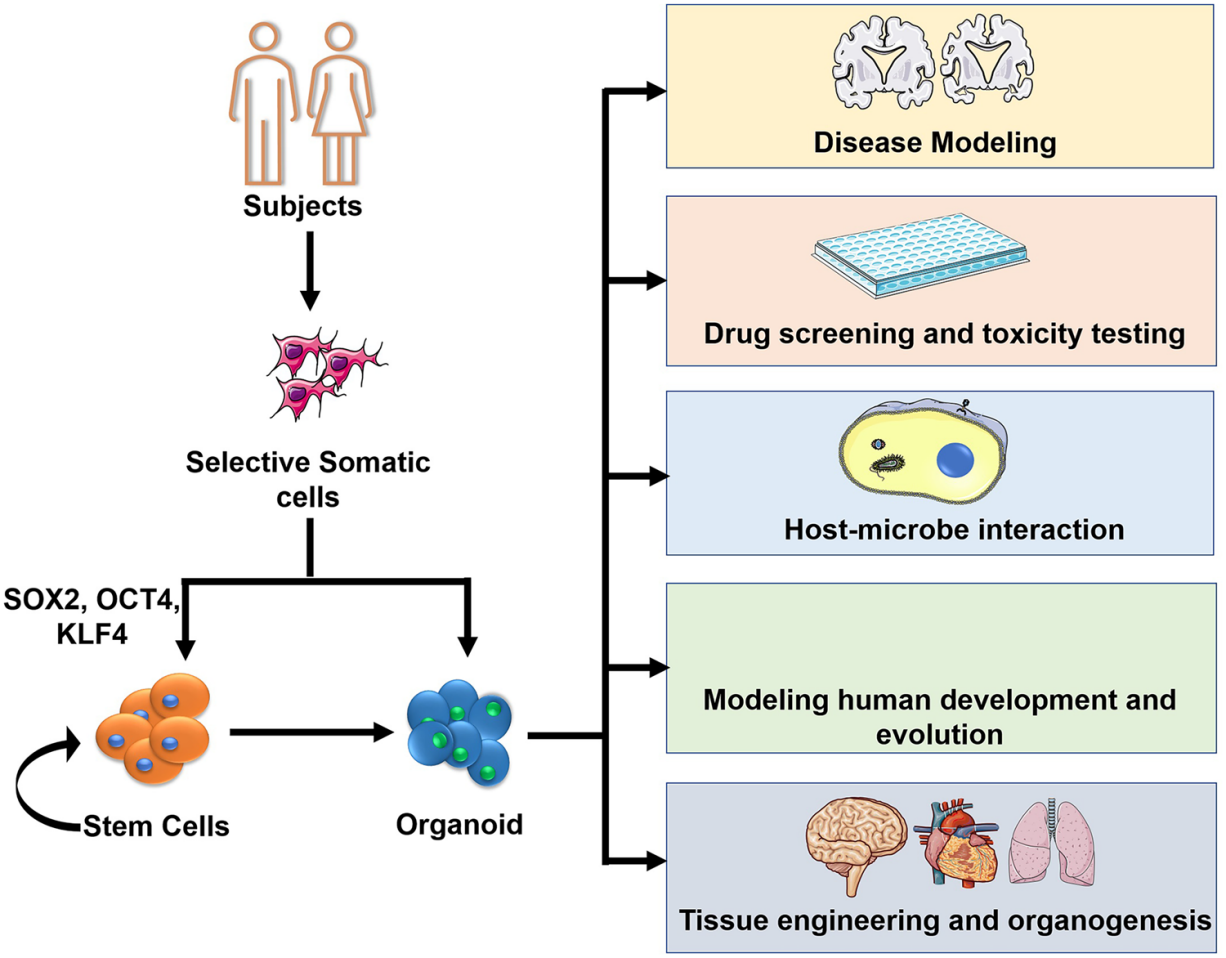
Applications of patient-derived organoids in personalized medicine

With advances in these techniques, the future could see highly reproducible lab-on-chip models that could be used for high throughput analysis. Making these systems more uniform will allow for data generated through experiments to be reproducible. With the advent of personalized medicine, the organoid-on-chip models could be used in personalized drug testing in clinical settings. They could also help in clinical trials by abrogating the “false positive” drugs that may get through the screening system due to variations in animal models and monoculture settings. Figure 3 highlights the important applications of patient-derived human organoids in developing personalized medicine.

## Application of organoids in evolutionary studies

Brain organoids have been used to understand the changes that occurred during the evolution of man from Denisovan and Neanderthals who are the extant closest relatives to modern humans. One method described by Trujillo et al. (2021) uses introducing a Denisovan/Neanderthal gene variant in the organoids via hPSCs, and observing the morphological, developmental and synaptogenic changes, if any, compared to modern human cortical organoids as controls. The authors first completed a genome wide analysis and identified 61 gene coding variants observed in Denisovan or Neanderthal genomes that carried no substitutions in humans. *NOVA1*, neuro-oncological ventral antigen 1, one of the candidate gene showed an isoleucine-to-valine single nucleotide substitution at position 200 in archaic genomes (Denisovan/Neanderthal) which was lost in humans during evolution. The hPSCs used for the generation of cortical organoids were CRISPR-Cas9 genome edited and the human *NOVA1*^*Hu/Hu*^ gene replaced with the archaic *NOVA1*^*Ar/Ar*^. There was an observed increase in the rigid surface complexity, slower growth, and altered electrophysiological properties in cortical organoids of *NOVA1*^*Ar/Ar*^ archaic variant as compared to *NOVA1*^*Hu/Hu*^ human cortical organoids. This study revealed the mutations were exclusively found in the modern day humans. The human-specific mutations in *NOVA1*^*Hu/Hu*^ not observed in archaic *NOVA1*^*Ar/Ar*^ may be due to functional role during the course of evolution. Various researchers, although commended this work, have been skeptical due to two reasons: a) there is a limitation to validate these evolutionary claims in the not well-preserved soft tissues of Neanderthal brains and b) the organoid may not represent the actual Neanderthal brain, moreover, the observed difference in organoid morphology by this single protein change to archaic one would be due to compounding effect (Remmel 2021).

## Limitations of organoid systems

Despite the potential of organoids in understanding disease and development, like any other system, there are limitations that need to be addressed. First, although organoids have been demonstrated to mimic the cellular microenvironment of tissues more faithfully than 2D culture systems, there are minute differences in gene expression patterns between organoids and the corresponding in vivo tissues (Pollen et al. 2019). Research is still on to determine the extent to which the organoids can recapitulate the in vivo organ systems (de Souza 2018). Organoid formation should ideally mimic the organ development in its entirety as in the embryo/fetus, but the timing and concentration of various growth factors involved makes it difficult to generate the organoids that match the organs exactly (Kim et al. 2020). Another challenge being that organoids generated from hPSCs, show fetal tissue gene expression profiles, which may not be ideal to study the mature tissue or organ systems (de Souza 2018). Organoids derived from primary tissues can be contaminated by cells from neighboring tissues depending on the sampling method. It has also been reported that 25–95% of tumoroids were shown to be contaminated by “normal” cells (Pauli et al. 2017). Due to the complexities of organoids compared to that of 2D monolayers, tackling the variability could become a tedious task. To start with, the hPSCs used for generating spheroids show variability in their genotypes. Secondly, the batches of organoids from such starting material would show further differences and thirdly this variability also percolates down to multiple organoids of the same batch and within the regions of the same organoids (de Souza 2018). These heterogeneous cultures will contain various clones, with some clones being more predominant than others. Thus, the proportion of cells in an organoid culture harboring a mutation of interest will have to be validated to accurately determine drug response and make experiments reproducible (Huch et al. 2017). These challenges add to further woes with the variability in the growth conditions of different batches that could skew results and reproducibility of experiments (Huch et al. 2017). Furthermore, the lack of vasculature along with immune cells makes it difficult to recapitulate the same conditions as in the tissue/organ systems in vivo (de Souza 2018).

Growth of organoids in Matrigel, a tumor-derived ECM matrix, also impedes their potential clinical application due to the risk of transfer of pathogens and immunogens (Clarke et al. 2018). Growth of organoids in synthetic hydrogels have shown promise in dealing with this limitation, with the same hydrogels used as vehicles to deliver the organoids in mice models (Cruz-Acuña et al. 2018). Generation of organ and tissue specific macro-architecture in organoids remains another challenge that hinders their use in transplantation therapies (Grebenyuk and Ranga 2019). Advances in the development of 3D scaffolds that accurately mimic in vivo tissue architecture will lead to more success in organoid transplantation strategies.

As organoids are found to have more and more relevance and applications, large scale manufacture of organoids becomes inevitable. Due to heterogeneity of cell types, generation of viable organoids with higher yield has been a major challenge. For instance, while generating the intestinal organoids, not all the aggregate of cells bud into organoids. However, by selectively culturing the aggregate of cells that has the potential to develop into organoids, would increase the yield in cultures. Arora et al. (2017) developed a technique of the hind gut cell that can aggregate and can grow into the intestinal organoids. They used an automated capillary-based sorting system where an image processing and analysis unit selects the viable spheroids by identifying them based on the given preset characteristics such as morphology, size, number of cells etc., via a microscope, followed by a three-axis (x, y and z axes) positioning unit that renders micropipette in place along with the spheroid harvesting unit that harvests the potential spheroids for further use.

Ethical challenges in organoid culture are related to the use and derivation of PSCs. Informed consent of the donor in case of patient-derived organoids for further use and limitations of their applications have been considered in the organoid culture research similar to those in developmental biology and clinical research (Munsie et al. 2017).

## Conclusion

This review has aimed to collect information on the various strategies employed in organoid culture and to address the potential of this form of cell culture in modeling diseases, as a tool for drug testing, and to better understand the developmental processes involved in the formation of various organs in the human body. The ability of organoids to recapitulate the genetic and epigenetic signatures of the original tissue is of relevance in the field of personalized medicine and can help find therapies against various diseases and conditions. Methods to co-culture tumoroids with tumor environmental cells have allowed the study of tumor-environment interactions, which could possibly lead to a better understanding of the progression of cancer and its potential treatment. The combination of organoid culture with CRISPR/Cas technology and organ-on-chip devices to incorporate and study vasculature are methods that may help better recapitulate in vivo tissue biology*.* Organoids derived from patients do not deviate in their phenotype, and thus are good models to study disease. Furthermore, organoid biobanks hold the promise of helping us understand the contributions of genetic variations to drug response, helping improve existing therapies and discover new ones. Thus, organoids hold the promise of bridging the gap between animal model systems and human subjects. In the future, this technology may help reduce the use of animals in in vivo drug testing, provide an ethical alternative to animal testing. Use of organoids in evolutionary biology, may additionally serve along with the conventional methods of fossil studies and computational biology.
